# Development of Cutting and Suction Device with Twist Blade Screw for Minimally Invasive Surgery: Evaluation of Suction Performance

**DOI:** 10.1371/journal.pone.0131931

**Published:** 2015-07-01

**Authors:** Yusuke Fujii, Takashi Suzuki, Manabu Tamura, Yoshihiro Muragaki, Hiroshi Iseki

**Affiliations:** 1 Graduate School of Medicine, Tokyo Women’s Medical University, Tokyo, Japan; 2 Medical Device Strategy Institute, Japan Association for the Advancement of Medical Equipment, Tokyo, Japan; 3 Institute of Advanced Biomedical Engineering and Science, Tokyo Women’s Medical University, Tokyo, Japan; 4 Faculty of Advanced Science and Engineering, Waseda University, Tokyo, Japan; 5 R&D Department, J. Morita MFG. Corp., Kyoto, Japan; Irvine, UNITED STATES

## Abstract

In this study, we aim to develop a narrow-diameter and long-bore device for minimally invasive surgery that achieves the simultaneous cutting and suction of body tissue such as the diseased part of an organ. In this paper, we propose a screw made of a thin metal plate, and we developed a prototype device using this screw. For smooth operation, the suction performance must be superior to the cutting performance. Therefore, we performed experiments and evaluated the suction performance of the developed device assuming the crushed tissue pieces correspond to a highly viscous fluid. From the results, we confirmed that the suction volume is almost proportional to the rotation speed of the screw in the low speed range, and the device has an upper limit of suction volume at a certain rotation speed. Considering practical use, its proportional speed range is suitable for the device controllability of cutting and suction volume, and the size of the device tip needs to be 1 mm or more. Based on these conditions, we are planning to examine the shape of the cutting edge for realizing efficient cutting and suction and we will complete the device.

## Introduction

Minimally invasive surgery has come into use in a wide range of fields in recent years, with the aim of improving patients’ quality of life. Surgical instrumentation has kept pace with this, with the development and use of instruments such as narrow-diameter, long-axis forceps and electric scalpels, which are widely used in neurosurgery, for example [1].

The use of minimally invasive surgery can minimize physical and mental stress on the patient. On the other hand, the burden on the surgeon is very large because precise work is required in a limited space. For example, if removing lesions using a device such as narrow-long forceps, since resectable tissue mass in a single operation is small, it is necessary to remove tissue in many separate operations or to use a long, narrow suction tube. However, narrowing the diameter and increasing the length of negative pressure suction instruments are known to greatly reduce their suction performance. For these reasons, the increased operation time is a particular problem.

In order to improve the efficiency of minimally invasive surgery, it has been desired to develop a device for realizing the cutting, transport, and discharge simultaneously. For this purpose, devices have been developed as surgical instruments that are capable of simultaneously resecting and removing body tissue, such as the CUSA system (Integra LifeSciences Corp., New Jersey, USA) [2] and Sonopet (Stryker Corp., Michigan, USA) [3]. Furthermore, research and development, of devices such as one combining a CO_2_ laser and a negative pressure suction tube [4] and a suction tube with an electric scalpel function in itself [5] has been performed. These devices also use negative pressure as their suction method. CUSA and Sonopet break down tissue into tiny pieces and suction them simultaneously with the injection fluid also used for cooling. This construction helps avoid clogging and loss of suction performance. Another two devices are also possible to lower the viscosity by sucking the target tissue with water; however, it is insufficient to solve the fundamental problems of clogging.

For these reasons, in this study, we develop a narrow-diameter, long-bore device capable of efficient cutting and suction without becoming clogged. In this paper, we investigated the mechanism for realizing cutting and suction. In addition we developed a prototype device and evaluated its performance.

## Methods

### Narrow-diameter, long-bore cutting and suction device

#### Investigation of methods to achieve cutting and suction simultaneously

Outside of the medical field, screw mechanisms are widely used for foods such as minced meat or pastes, and for the transport of highly viscous fluid substances such as resins and cement [6, 7]. Attempts have been made to adapt this design to the field of ear, nose, and throat (ENT) medicine with the aim of suctioning highly viscous exudate in cases of exudative otitis media [8, 9], and the screw mechanism has been successful in suctioning highly viscous fluids that would be difficult to aspirate with an extremely narrow-diameter negative-pressure suction pipe.

As the screw undergoes the physical rotation, it is possible to achieve the avoidance and elimination of clogging during suction. At the same time, it is also possible to use the rotary motion for the cutting by an edged screw tip.

In this study, we similarly adopted a screw construction in the prototype device. Cutting and suction performance of the device is determined by the shape of the screw and the rotation speed. Especially in the case in which the suction performance is inferior to the cutting performance, suction causes disruption. First of all, we should be clear about the suction performance.

However, analysis of the flow with the screw is very difficult [10, 11], Roberts [12] has formulated the flow volume by the screw conveyor targeting for the bulk solid, but the viscosity was not considered. Thus, suction performance must be clarified by experiments; in this paper, we evaluate the suction performance of the prototype device with a screw construction.

### Device structure

#### Screw construction

**Fig 1:**
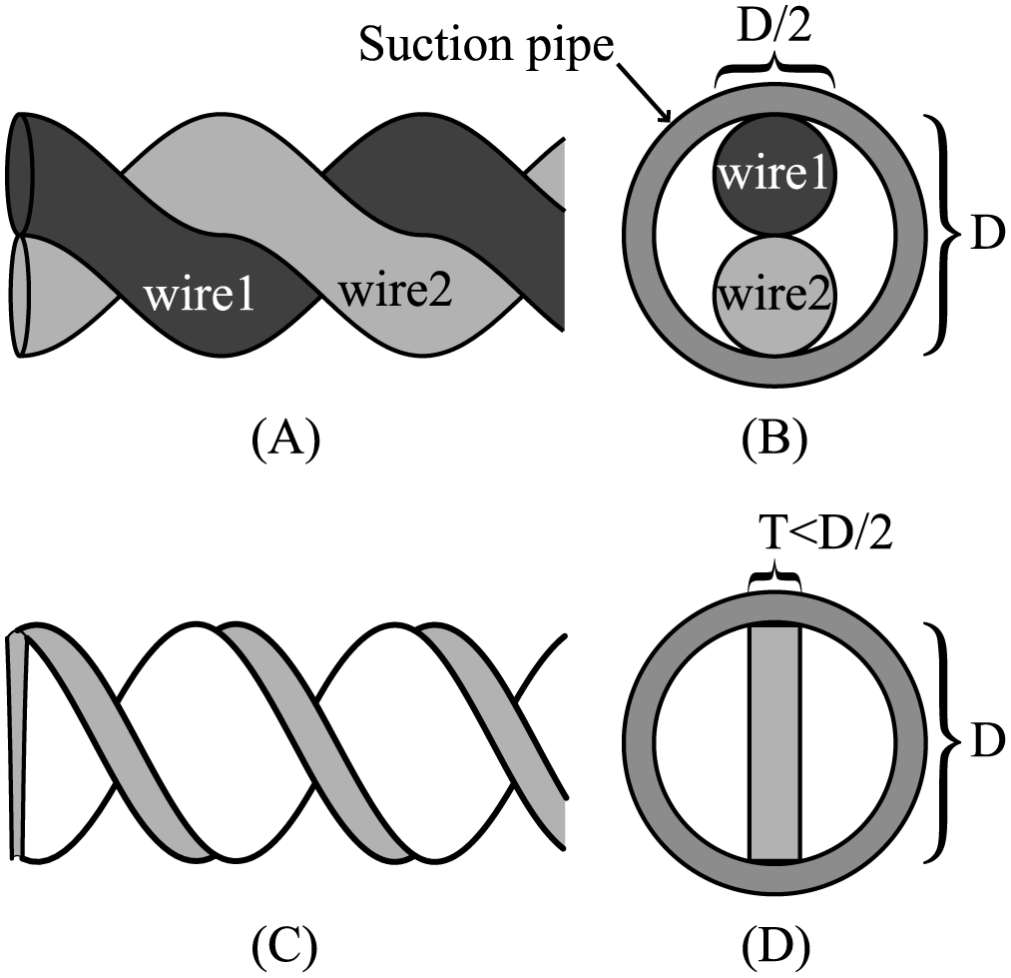
Comparison of the screw shape and a cross-sectional view of the suction device tip. (A)Shape of double wire screw made of two wires. (B)A cross-sectional view of the suction pipe on double wire system. (C)Shape of twist blade screw made of single plate. (D)A cross-sectional view of the suction pipe on twist blade system.

The maximum length of narrow screws that consist of a helical shape surrounding a rotational axis, e.g. 1 mm diameter screws, is only about a dozen millimeters because of processing limitations. Therefore, the long-axis, narrow-diameter screws required for this project are technically difficult to produce. On the other hand, the rotational suction device for ENT use [8, 9] shown in Fig 1(A) is made by twisting together two very fine wires with a diameter of 0.15 mm to form a screw 0.3 mm in diameter. In order for the screw to rotate in a stable manner and create suction, it must slide against the inner wall of the suction pipe, as shown in Fig 1(B). Structurally, the transport space thus accounts for 50% of the cross-sectional area of the suction pipe, and high efficiency cannot be expected.

In this study, we addressed this issue by producing a “twist blade screw.”

As shown in Fig 1(C), the twist blade screw was made by twisting a thin metal plate to form a helical structure. As seen in Fig 1(D), the twist blade screw could provide a wider transport space compared with a double wire screw. Moreover, because a twist blade screw is constructed from a single thin plate, it is able to process a cutting edge.

#### Prototype device

**Fig 2:**
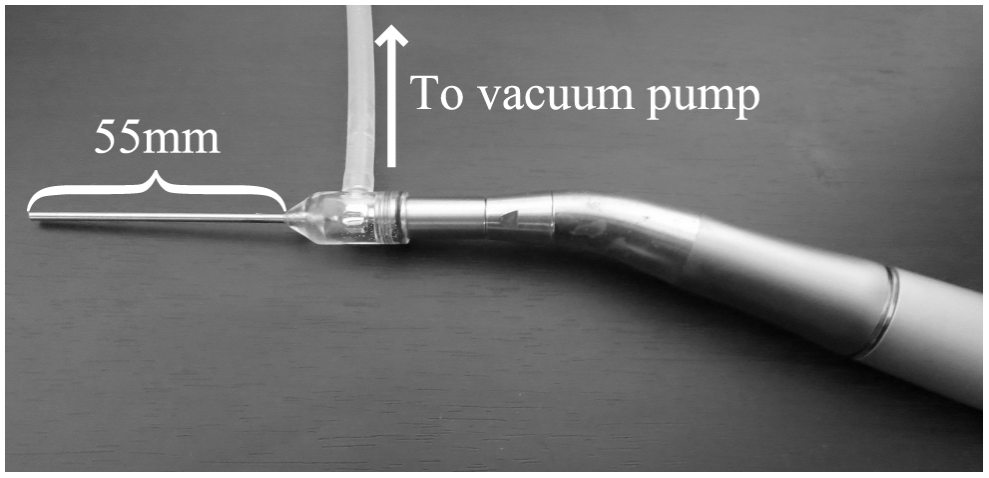
External view of prototype device.

Fig 2 shows our prototype device.

This device was based on a dental cutting instrument. It is equipped with an internal gear, which transmits rotation supplied by a motor drive to the screw at the tip. It is also constructed of a two-gear system that can increase the rotation speed by up to approximately five times.

Most regular suction pipes are around 100 mm long, including the portion gripped by the operator, and the length of the small-diameter portion is approximately 50 mm. We used this as a reference for the length of the suction pipe in our prototype device, which was set at 55 mm to allow a degree of leeway.

**Fig 3:**
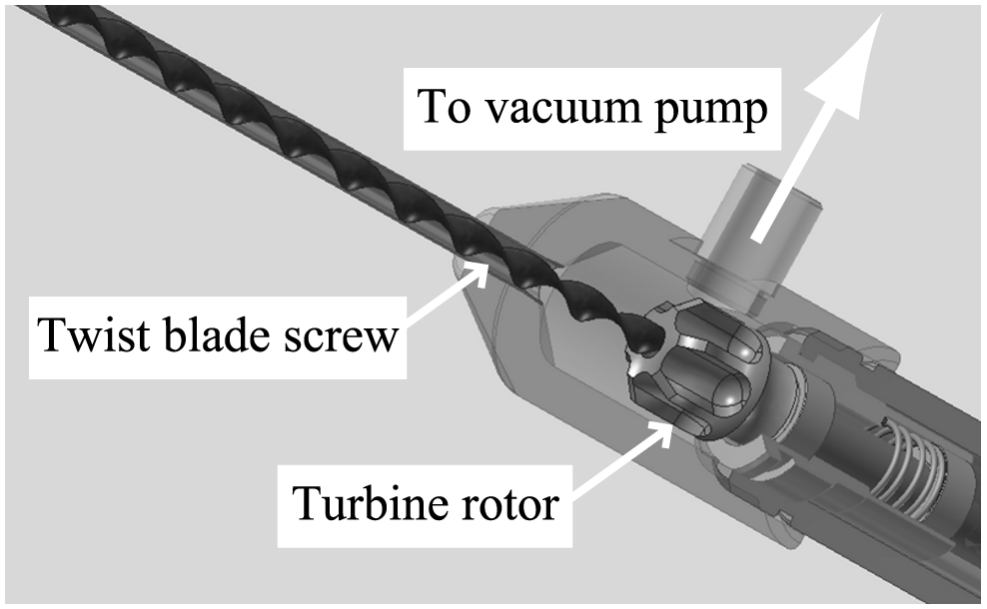
Scheme of internal structure of prototype device.

In order to expel the substance being suctioned and transported into the device, we provided a drainage port with an internal diameter of 4 mm that could be attached to a vacuum pump. As shown in Fig 3, we also added a spur-gear shaped turbine rotor to the base of the screw, to eject the substance effectively in the direction of the drainage port as the screw rotated. We confirmed that, during high-speed rotation, some negative pressure was generated by the turbine rotor, but this was negligibly small compared with the pressure of the drainage vacuum, and the suction effect was small.

**Fig 4:**
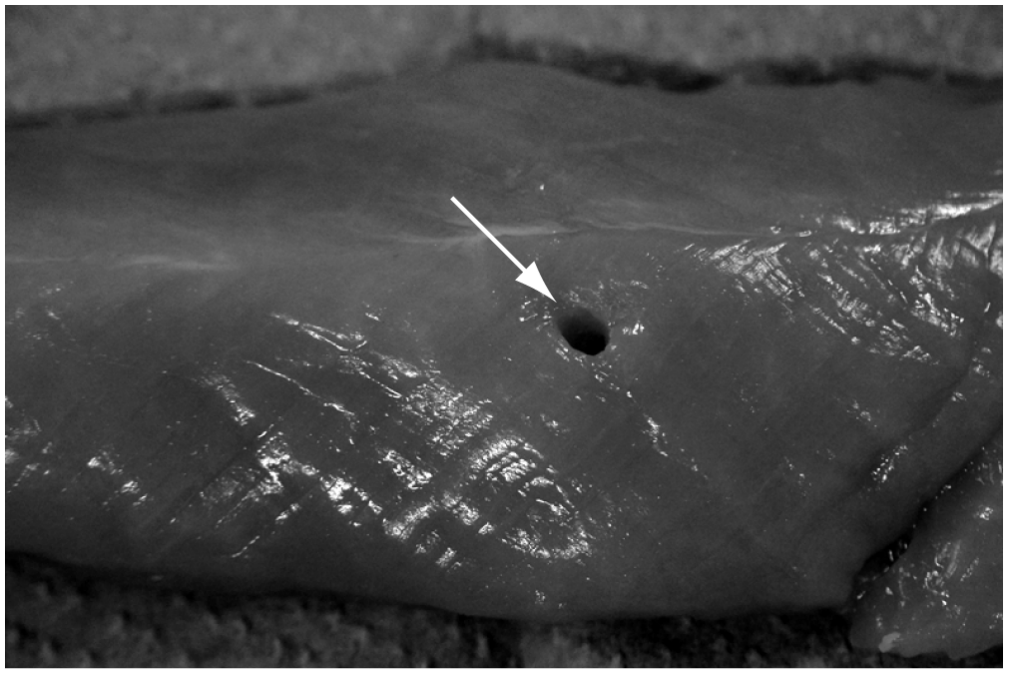
Fresh chicken cut and suctioned after verifying the performance of prototype device.

We verified the performance of prototype device using fresh chicken meat as a target and confirmed that cutting and suction of biological tissue can be realized (S1 Video, Fig 4).

### Performance evaluation experiments

In order to evaluate the suction performance of the device, we investigated the association between the screw rotation speed and the suction amount.

#### Experiment environment

Given that soft solids in paste form behave in a similar way to highly viscous fluids [13, 14], in these experiments we used standard liquids for calibrating viscometers used for industrial purposes as the target fluids for suction. The experiment materials comprised water and four different standard viscosity liquids (Nippon Grease Co., Ltd.). Table 1 shows the viscosity value of each liquid, together with the viscosity values of human blood, fish sausage [13] and meat mixture [14] as a reference.

**Table 1:** Viscosity value of the target liquids, blood, fish sausage and meat mixture.

Target liquid	Viscosity value(20°C) [mPa·sec]
water	1
JS2000	1,800
JS14000	12,000
JS52000	46,000
JS160000	140,000
blood	3–5
fish sausage [13]	3,000–30,000(15°C)
meat mixture [14]	> 200,000(15°C)

A dental brushless motor (TORX TR-91, J.Morita MFG. Corp., Kyoto, Japan) with a maximum rotation speed of 40,000 rpm and a dedicated controller were used as the drive sources of the device. The multiplying gear system fitted to the prototype device enabled the maximum rotation speed to be increased to 200,000 rpm. The controller enabled the internal control of rotation and torque, but we suppressed these functions and used only the simple ON-OFF switch and manual control of rotation speed.

An electronic balance (FX-120i, A&D Co., Ltd., Tokyo, Japan) capable of measuring to an accuracy of 0.001 g was used to measure the amount of target liquids before and after suction, and the suction volume was calculated from the accumulation using the density.

A medical vacuum pump (maximum negative pressure -70 kPa) was used to expel the suction target liquids transported to the inside of the device. A silicon tube (internal diameter 4 mm, external diameter 6 mm) was attached to the drainage port in the device shown in Fig 2, and connected to the vacuum attachment via an effluent trap.

Table 2 shows the relationships between the tip diameter of the device and the size of the twist blade screw. A blade thickness of 0.1 mm was used for the twist blade screw in order to provide sufficient strength. The blade width was determined in accordance with the thickness and the internal diameter of the tip pipe. The twist pitch (interval) was the minimum possible that could be processed.

**Table 2:** Dimensions of twist blade screw related to the tip diameter of device.

Tip diameter(inner/outer) [mm]	Blade thickness [mm]	Blade width [mm]	Twist pitch [mm]
0.3/0.5	0.1	0.24	0.8
0.5/0.8	0.1	0.44	1.3
0.8/1.0	0.1	0.76	2.8
1.8/2.0	0.1	1.76	7.8
2.8/3.0	0.1	2.76	14.9

#### Experimental methods

Graded screw rotation speeds of the device were set, suction was applied to the target liquid at the set rotation speed, and the weights before and after the suction time were measured and recorded. Measurements were performed ten times, and the mean changes in weight were regarded as the suction amount at the set rotation speed. During the experiments, the device was fixed and suction was applied in such a way that the tip was perpendicular to the surface of the liquid.

However, if the rotation speed decreased by more than 10% from the set rotation speed due to the load during suction, this was regarded as mechanical over-load and no further measurements were performed.

**Fig 5:**
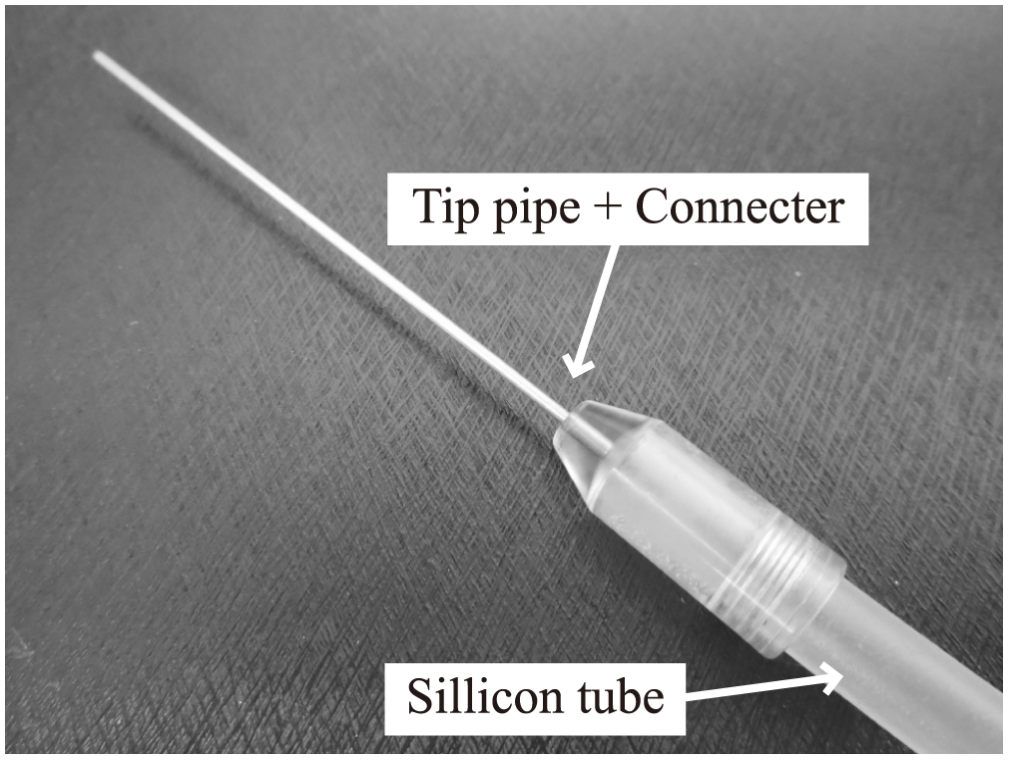
Simplified vacuum prototype.

We also made a simplified vacuum (Fig 5) using the same pipes that were used at the tip of the device. These were directly connected to a vacuum pump and the same experiments were performed, after which the suction volume were compared.

In preparation for the experiments, the target liquids were allowed to stand for an adequate time in a room maintained at room temperature (15°C). External disturbances such as changes in the internal resistance of the suction line before and after the application were also eliminated by applying suction to the target liquid so that it filled the entire line before the start of each experiment.

## Results

**Fig 6:**
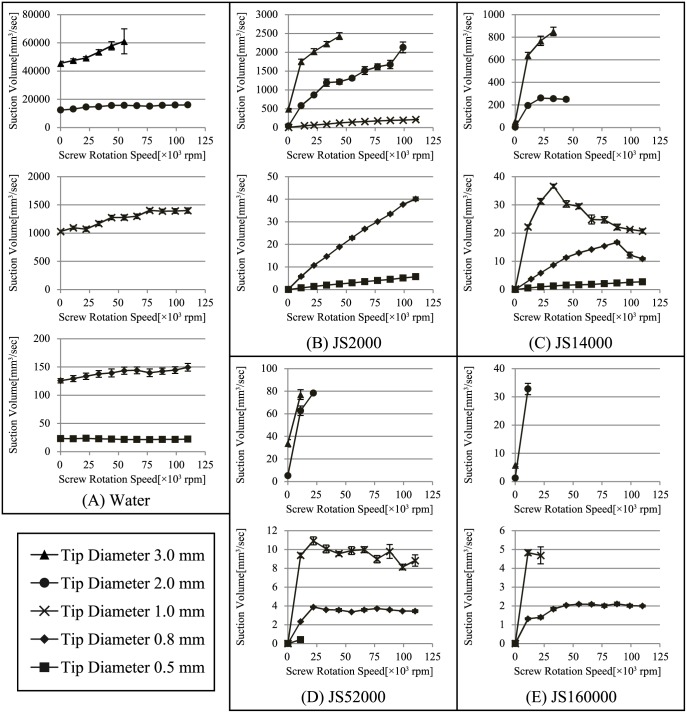
Plot of screw rotation speed vs. suction volume. Target liquid is (A)water, (B)JS2000, (C)JS14000, (D)JS52000, (E)JS160000. The horizontal axis indicates the screw rotation speed and the vertical axis indicates the volume suctioned per unit time. Each data plot represents the averaged measurement value and the error bars represent the standard deviation. The 0 rpm plot shows the volume as a result of the supplementary suction generated by the vacuum pump for discharge of the liquid suctioned.

Table 3 and Fig 6 show the experimental results.

**Table 3:** Suction performance comparison between vacuum and prototype device.

Target liquid	Tip dia. [mm]	Suction volume(mean ±SD(%)) [mm^3^/sec]			Device(Max) / Vacuum
		Vacuum	Prototype device		
			0 rpm(Supplementary)	Maximum value	
water	0.5	172.3 ±2.1%	23.08 ±4.6%	23.6 ±4.7%	0.14
	0.8	758.08 ±2.4%	125.62 ±3.1%	149.42 ±4.5%	0.20
	1.0	4,131.7 ±2.4%	1,028.2 ±2.5%	1,402.6 ±2.3%	0.34
	2.0	31,078 ±2.3%	12,369.8 ±1.3%	16,003.4 ±2.5%	0.51
	3.0	88,719.5 ±2.6%	45,499 ±3.0%	61,077.5 ±14.4%	0.69
JS2000	0.5	0.47 ±25.4%	0	5.65 ±0.9%	12.02
	0.8	1.34 ±10.9%	0.17 ±11.7%	40.16 ±2.1%	29.97
	1.0	10.45 ±8.5%	2.82 ±5.3%	211.22 ±1.7%	20.21
	2.0	430.81 ±2.6%	38.90 ±2.5%	2,129.97 ±6.8%	4.94
	3.0	1,990.91 ±5.2%	489.89 ±3.5%	2,428.54 ±3.7%	1.22
JS14000	0.5	0	0	2.72 ±2.6%	∞
	0.8	0.23 ±19.6%	0	16.74 ±2.3%	72.78
	1.0	1.08 ±10.4%	0.22 ±23.2%	36.60 ±1.9%	33.89
	2.0	121.59 ±7.8%	3.55 ±3.3%	261.10 ±4.8%	2.15
	3.0	604.18 ±2.1%	53.93 ±4.8%	851.24 ±4.4%	1.41
JS52000	0.5	0	0	0.41 ±6.8%	∞
	0.8	0	0	3.88 ±1.8%	∞
	1.0	0.46 ±15.8%	0	10.92 ±3.9%	23.73
	2.0	17.53 ±4.2%	5.23 ±7.5%	78.35 ±0.7%	4.47
	3.0	58.12 ±3.4%	33.83 ±9.6%	77.05 ±5.7%	1.34
JS160000	0.5	0	0	–	–
	0.8	0	0	2.11 ±4.2%	∞
	1.0	0	0	4.83 ±2.9%	∞
	2.0	11.45 ±6.6%	1.25 ±13.5%	32.79 ±6.1%	2.86
	3.0	36.83 ±3.3%	5.81 ±7.7%	–	0.16

Suction volume of the prototype device is indicated maximum measurement value in rotation speed range, shown in Fig 6.

The first point to note with respect to all the results is that the supplementary suction volume was less than that seen with the simplified vacuum with the same tip diameter. This may have been because the rotational axis was not completely enclosed, and the pressure was consequently reduced by the internal gaps.

A comparison of the results for water showed that the volume suctioned by the device was far less than that suctioned by a simplified vacuum with the same tip diameter, as can be clearly seen in Table 3. It can also be seen in Fig 6 that the increase in the suction volume in the measurement range of screw rotation speed was much less than the supplementary suction volume and the suction effect of the screw was small.

The experimental results for JS2000, shown in Fig 6(B), showed that except for tips of 2 mm diameter, the suction volume increased monotonically as the rotation speed increased, in a relationship that was almost directly proportional. With a 2 mm tip, the suction volume increased monotonically but not proportionally. A comparison with vacuum devices with the same tip diameter showed that the suction volume exceeded that of the vacuum device.

The experimental results for JS14000, shown in Fig 6(C), also demonstrated that the suction volume increased monotonically with 0.5 mm and 3 mm tips, while with the 0.8 mm tip the suction volume increased monotonically up to a speed of around 80,000 rpm, and then peaked at 88,000 rpm, after which the suction volume decreased. Similarly, with the 1 mm tip, the peak was seen at around 33,000 rpm and the suction volume decreased after 40,000 rpm. With the 2 mm tip, the peak was seen at around 22,000 rpm, after which the suction volume virtually leveled out. For all the devices, the volume exceeded that suctioned by a vacuum device with the same tip diameter at a low rotation speed.

The experimental results for JS52000, shown in Fig 6(D), showed that because of its very high viscosity, rotational resistance was extremely high, causing the screws to break when 0.5 mm tip was used at 22,000 rpm. With the 0.8 mm tip, the peak was seen at 22,000 rpm and the suction volume virtually leveled out. With the 1 mm tip, the volume becomes maximum at 22,000 rpm, and later it decreases gradually. With 2 mm and 3 mm tips, a decrease in rotational speed of more than 10% in the device is confirmed in the low rotation speed.

The experimental results for JS160000, shown in Fig 6(E), showed that the suction volume leveled out at approximately 30,000 rpm with the 0.8 mm tip. The screw of the 0.5 mm tip broke at 11,000 rpm and the 1 mm tip also broke but at 33,000 rpm. Similar to the result for JS52000, mechanical limits have been confirmed with 2 mm and 3 mm tips.

## Discussion

The experimental results showed that for the highly viscous liquids JS2000, JS14000 and JS52000, the suction performance of all size devices exceeded that of vacuum devices with the same tip diameter. With the exception of the case where the 3 mm tip was used, similarly high levels of suction performance were also seen for JS160000.

The difference in suction performance between our device with a 3 mm tip and a vacuum device was smaller than the corresponding differences in performance for other tip sizes. This may have been caused by a structural issue: the wider the diameter, the greater the rotational resistance due to friction between the screw and the inner diameter of the pipe. In our experiments, we set the rotation speed of the device to a constant level and did not control it in line with the load, which made it easy for the rotation speed to decrease. There may thus be room to improve the performance of the device by adjusting the control system.

Our results showed, however, that the twist blade screw is not very effective at suctioning low-viscosity fluids similar to blood. This may be because little friction is generated between low-viscosity fluids and the screw, as well as because the amount of fluid that cannot be properly transported increased due to effects such as leakage from gaps between the screw and the inside of the pipe. Due to the supplementary suction from the drainage vacuum, however, some suction is possible, although its suction performance is inferior to that of the vacuum device itself. Blood also poses the problem of its clotting action causing narrow-diameter suction tubes to become clogged, whereas in our device the rotation of the screw physically detaches clotted areas, reducing the problem of clogging. Should blood coagulate on the screw itself, however, this may decrease the suction amount, and further testing is therefore required.

We obtained the results for suction volume versus device size(screw pitch, pipe diameter), target viscosity and rotation speed. On the basis of these results, we estimated the experimental equation. The result of water and 0 rpm plots were excluded from estimation because the effect of the screw is very small.

Ignoring the effect of friction and viscosity, the average flow velocity *u*_*ave*_ is proportional to the screw rotation speed *N* and the head (energy) is proportional to *N* ^2^. Therefore, the suction volume *Q* is given by

$$Q=Su_{ave}=A\left(\frac{\pi d^{2}}{4}-td\right)pN,u_{ave}=ApN$$ (1)

where *S* is the effective cross-section, *A* is a constant value, *d* is the inner diameter of the tip pipe, *p* is the screw pitch and *t* is the screw thickness.

From Bernoulli’s principle concerning flow direction, the relationship between *u*_*ave*_ and *N* is expressed as follows,

$$\frac{u_{ave}^{2}}{2}+\frac{\Delta P}{\rho }+g\Delta L=B\frac{N^{2}}{2}$$ (2)

where Δ*P* is the pressure difference generated by the screw, *ρ* is the density of the target, Δ*L* is the flow path length and *B* is a constant. Considering the effect of friction, viscosity and other losses, Eq 2 is rewritten as

$$\frac{u_{ave}^{\prime 2}}{2}+\frac{\Delta P}{\rho }+g\Delta L=B\frac{N^{2}}{2}-\left(\lambda +C\right)\frac{u_{ave}^{\prime 2}}{2}$$ (3)

where $u_{ave}^{\prime }$ is the average flow velocity with viscosity resistance, *λ* is the friction coefficient and *C* is a constant. Therefore, from Eqs 2 and 3, when *λ* is assumed to be proportional to the dynamic viscosity *ν*, *u*′ *ave* is as follows,

$$u_{ave}^{\prime }=\frac{u_{ave}}{\sqrt{\left(C+D\nu +1\right)}},\;\lambda =D\nu$$ (4)

where C and D are constants.

**Fig 7:**
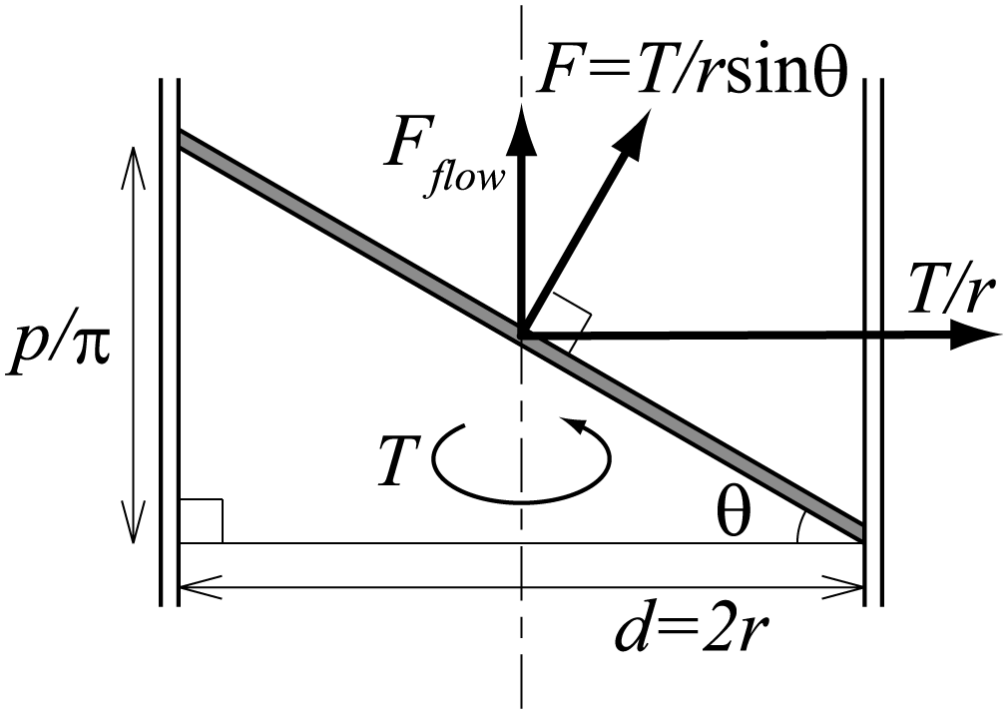
Force diagram.

As shown in Fig 7, the flow is generated by the force of the flow direction *F*_*flow*_. The relationship between the screw angle *θ* and *F*_*flow*_ at any given radius *r* is expressed by the following equation.

$$F_{flow}=F\cos\theta =\frac{T}{r}\sin\theta \cos\theta =T\frac{2\pi p}{{\left(2\pi r\right)}^{2}+p^{2}}$$ (5)

where *T* is the screw torque, *F* is the force applied to the target liquid. When the twist blade screw is assumed to have a constant twist angle in the radial direction, the sum of *F*_*flow*_ is as follows,

$$\int_{0}^{\frac{d}{2}}\;F_{flow}dr=T\;\int_{0}^{\frac{d}{2}}\;\frac{2\pi p}{{\left(2\pi r\right)}^{2}+p^{2}}dr=T\tan^{-1}\left(\frac{\pi d}{p}\right)$$ (6)

The suggested angle component ${\text{tan}}^{-1}(\frac{\pi d}{p})$ against input torque *T* serves to generate the liquid flow. Thus, from Eqs 1, 4 and 6, and considering the effect of supplementary suction pressure, the suction volume with viscosity resistance *Q*′ is expressed by the following equation.

$$Q^{\prime }=S\tan^{-1}\left(\frac{\pi d}{p}\right)\frac{pN}{\sqrt{C_{1}\nu +C_{2}}}+C_{3}$$ (7)

where *C*_1_, *C*_2_ and *C*_3_ are the values to estimate.

Using curve fitting with the least squares method, estimation was performed concerning the range of 50,000 rpm or less except for some of the plots apparently reduced. *C*_2_ and *C*_3_, however, would be dependent on the pipe diameter *d*, and the estimation was performed individually for each size of the device. In addition, the least squares method was processed using the standard deviations of the measured suction volume as weighting factors.

The estimation process consisted of three steps.

Step 1 Assuming *C*_3_ will be dependent on *d* and *ν* and the others will be constant, the relationship between *C*_3_, *d* and *ν* is estimated, e.g. *C*_3_ × *d*/*ν* + *C*_4_/*ν*, by repeating curve fitting.
Step 2 Assuming *C*_1_ will be independent of *d* and *C*_2_ will be constant, an approximate value is determined from the estimated value of *C*_1_ for each condition.
Step 3 Assuming *C*_2_ will be dependent on only *d*, the relationship between *C*_2_ and *d* is estimated and an approximate value of *C*_2_ is determined.

After repeating Step 1–3, each coefficient was estimated as shown in Table 4 and the empirical equation was obtained as follows.

$$Q=S\tan^{-1}\left(\frac{\pi d}{p}\right)\frac{pN}{\sqrt{C_{1}\nu +C_{2}/d+C_{3}}}+C_{4}\frac{d^{4}\sqrt{d}}{\nu }$$ (8)

**Table 4:** Estimated coefficients of Eq 8.

Tip diameter [mm]	*C*_1_	*C*_2_	*C*_3_ ±ASE(%)	*C*_4_ ±ASE(%)
0.5	0.006	23	≈ 0	100000
0.8	0.006	23	≈ 0	100000
1.0	0.006	23	1.98443 ±81.17%	100000
2.0	0.006	23	287.037 ±41.8%	100000
3.0	0.006	23	1258.73 ±23.25%	29465 ±5.8%

ASE is asymptotic standard error of estimated value.

As shown in Table 4, *C*_3_ is retained as a term dependent on each devices, and it will not be considered losses like heat loss. In addition, *C*_4_ of a 3 mm tip device is also retained. Since the difference of suction volume between the device with a 3 mm tip and the vacuum is about at most about a factor of 1.4, the work of the screw is relatively reduced. From the result with water, it is clear for low viscosity liquid that the relationship between suction volume of the vacuum *Q*_*vacuum*_ and of the screw *Q*_*screw*_ is *Q*_*vacuum*_ > *Q*_*screw*_ and *Q* = *Q*_*screw*_ + *Q*_*vacuum*_ can’t be established in such a case. Since the estimation had been performed by assuming that the effect of the screw is equal among the devices, it is probable that *C*_4_ of a 3 mm tip device was relatively reduced.

On the other hand, the graph from the results with a 0.8 mm tip for JS52000 and JS160000 is close to the shape of an asymptote in form. This means that it is possible that there is an upper limit for the flow velocity by viscosity resistance. Hence, the upper limit of the suction volume *Q*_*upper*_ would be depended on *d* and *ν* and expressed by these two parameters. The relationship between *Q*_*upper*_, *d* and *ν* was clarified by the same estimation process of Eq 8 and the following equation was obtained.

$$Q_{upper}=1.65\times 10^{6}\frac{\pi d^{2}}{4\nu }$$ (9)

**Fig 8:**
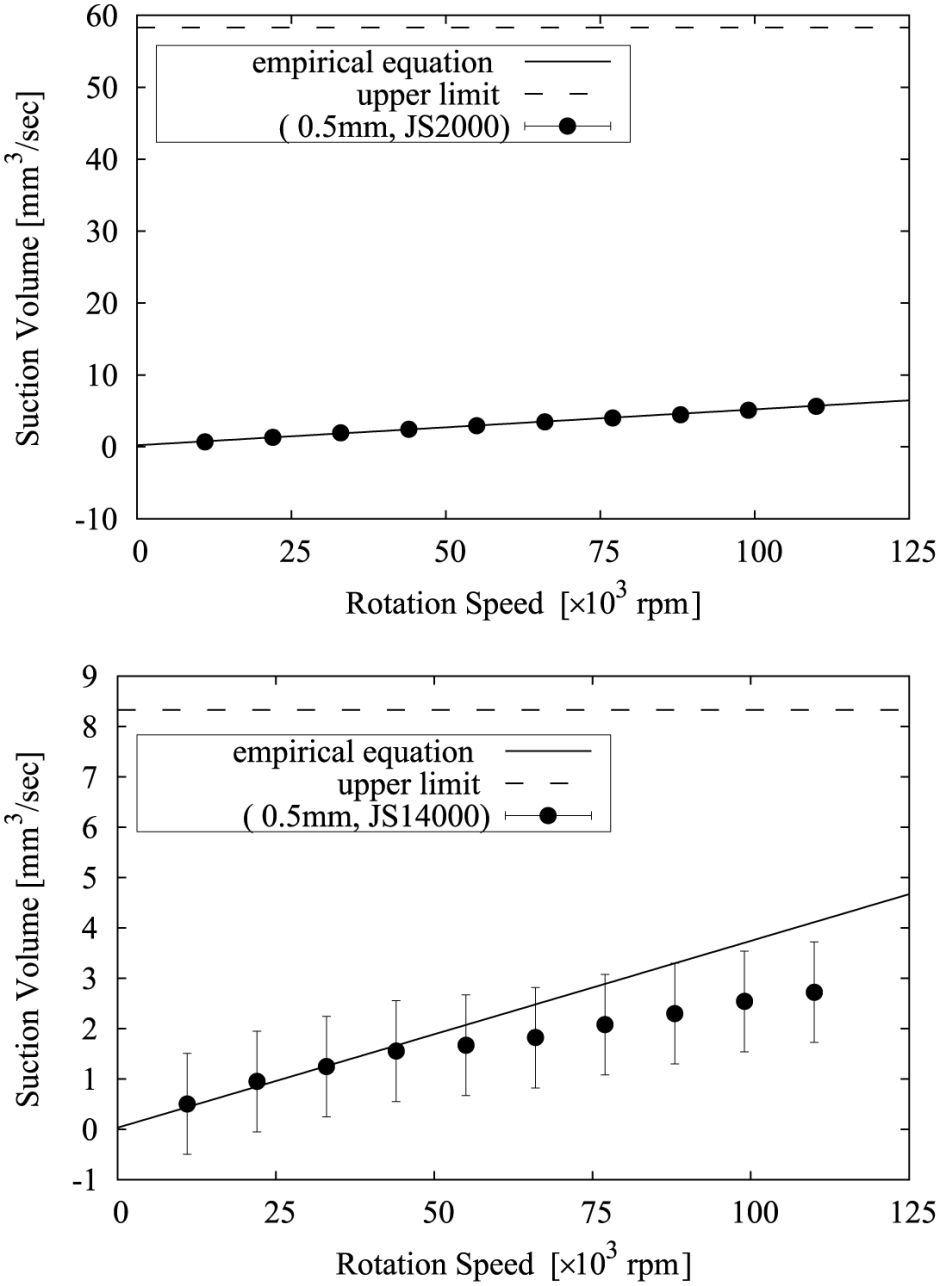
The relationship between Eqs 8 and 9 and the suction volume of the device with a 0.5 mm tip.

**Fig 9:**
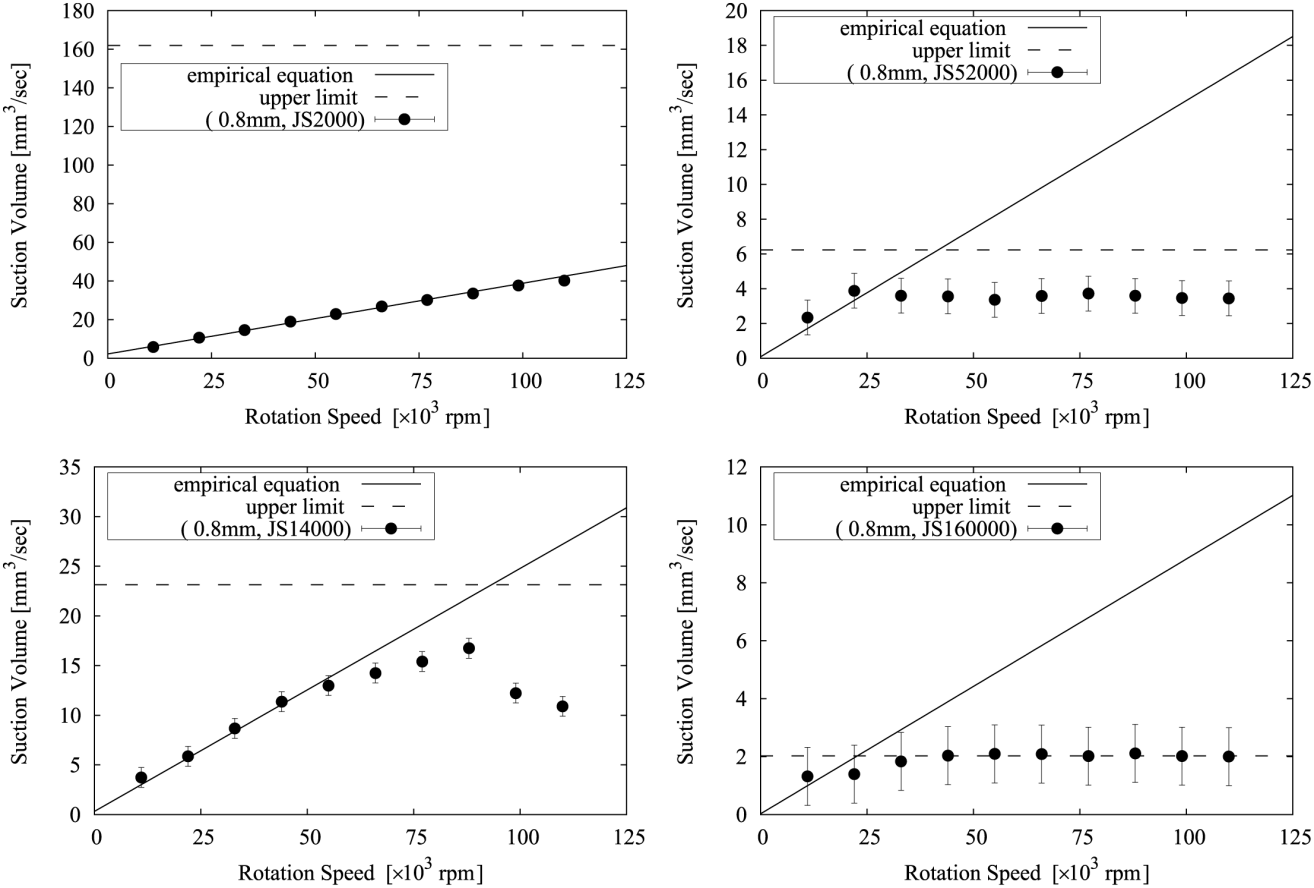
The relationship between Eqs 8 and 9 and the suction volume of the device with a 0.8 mm tip.

**Fig 10:**
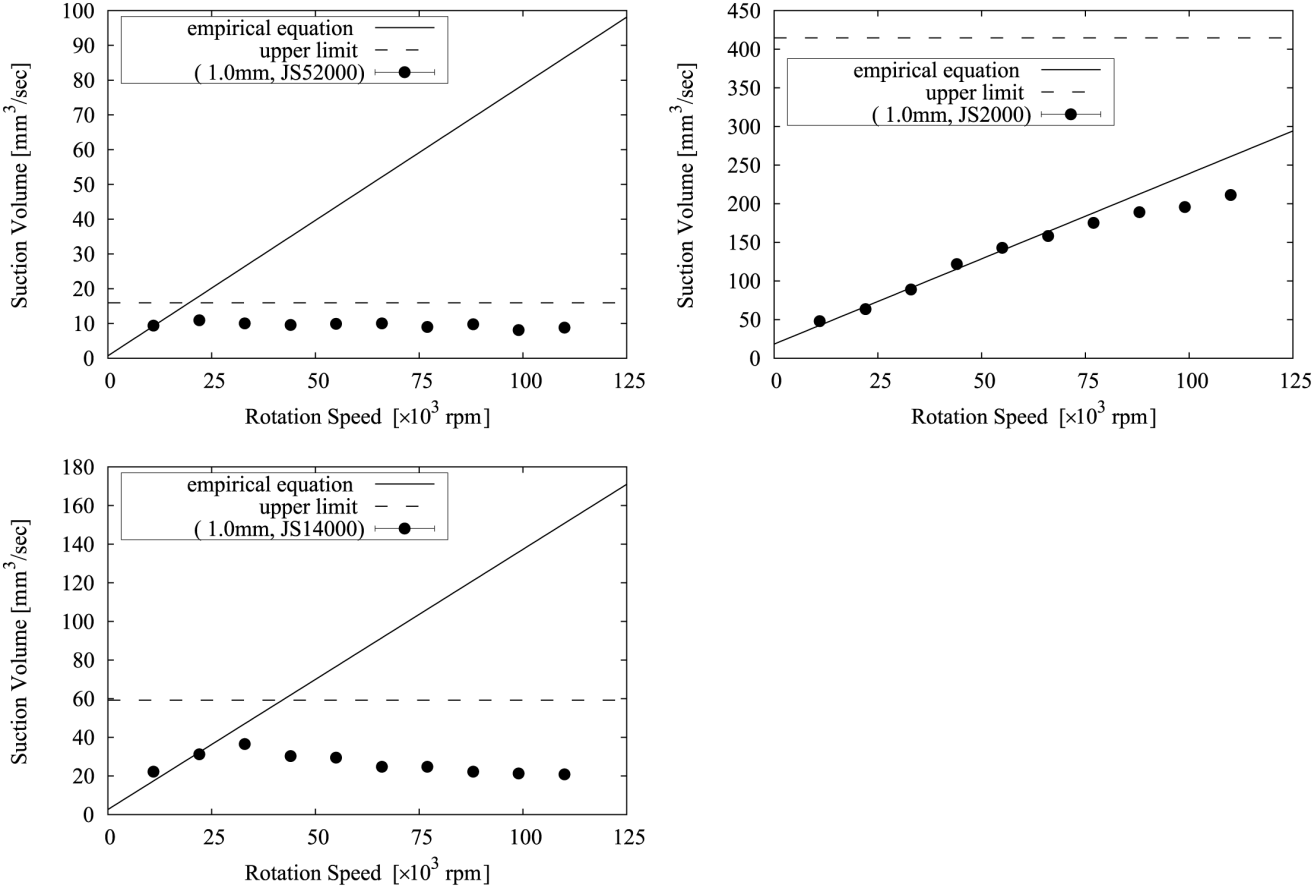
The relationship between Eqs 8 and 9 and the suction volume of the device with a 1.0 mm tip.

**Fig 11:**
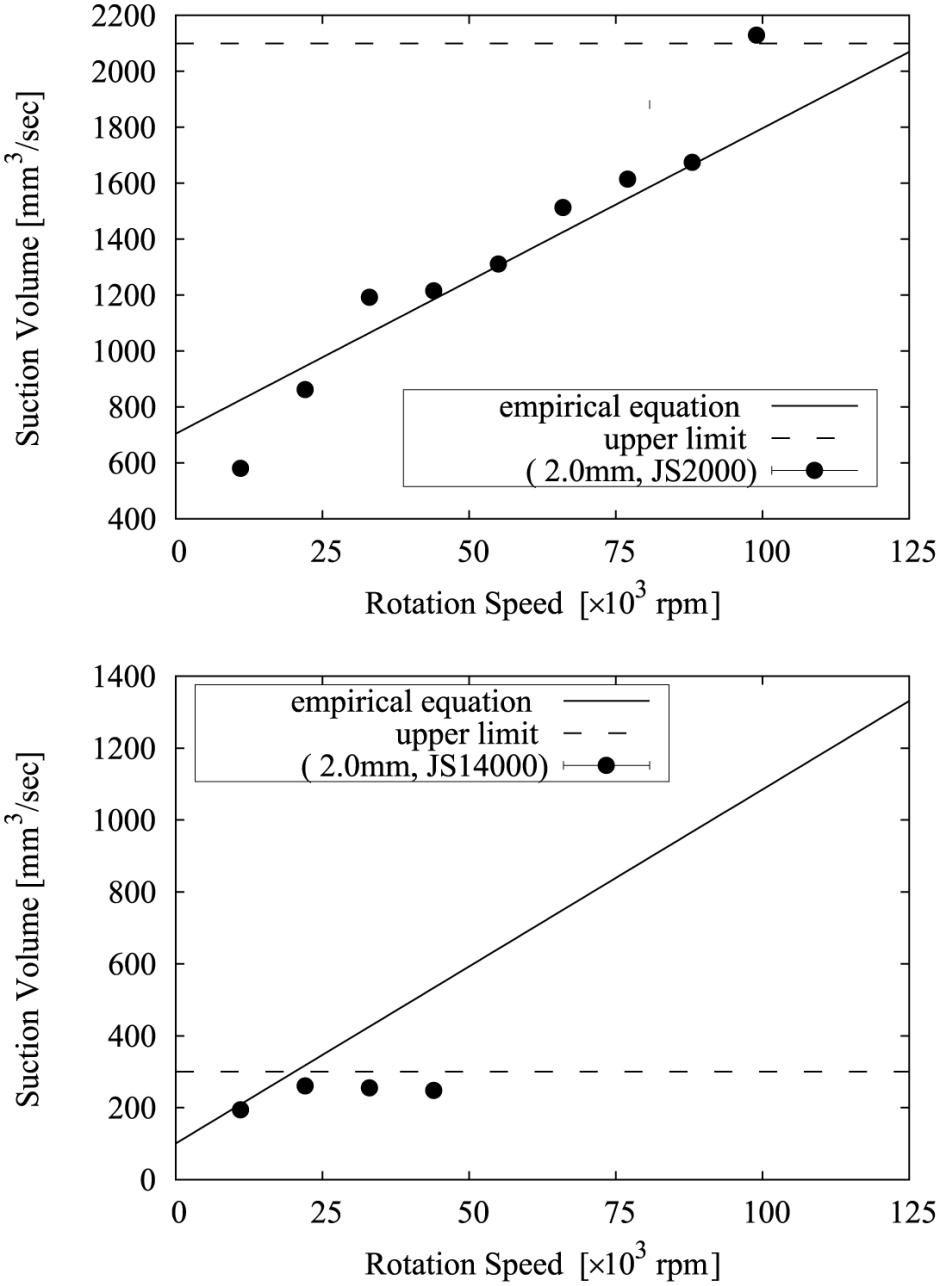
The relationship between Eqs 8 and 9 and the suction volume of the device with a 2.0 mm tip.

**Fig 12:**
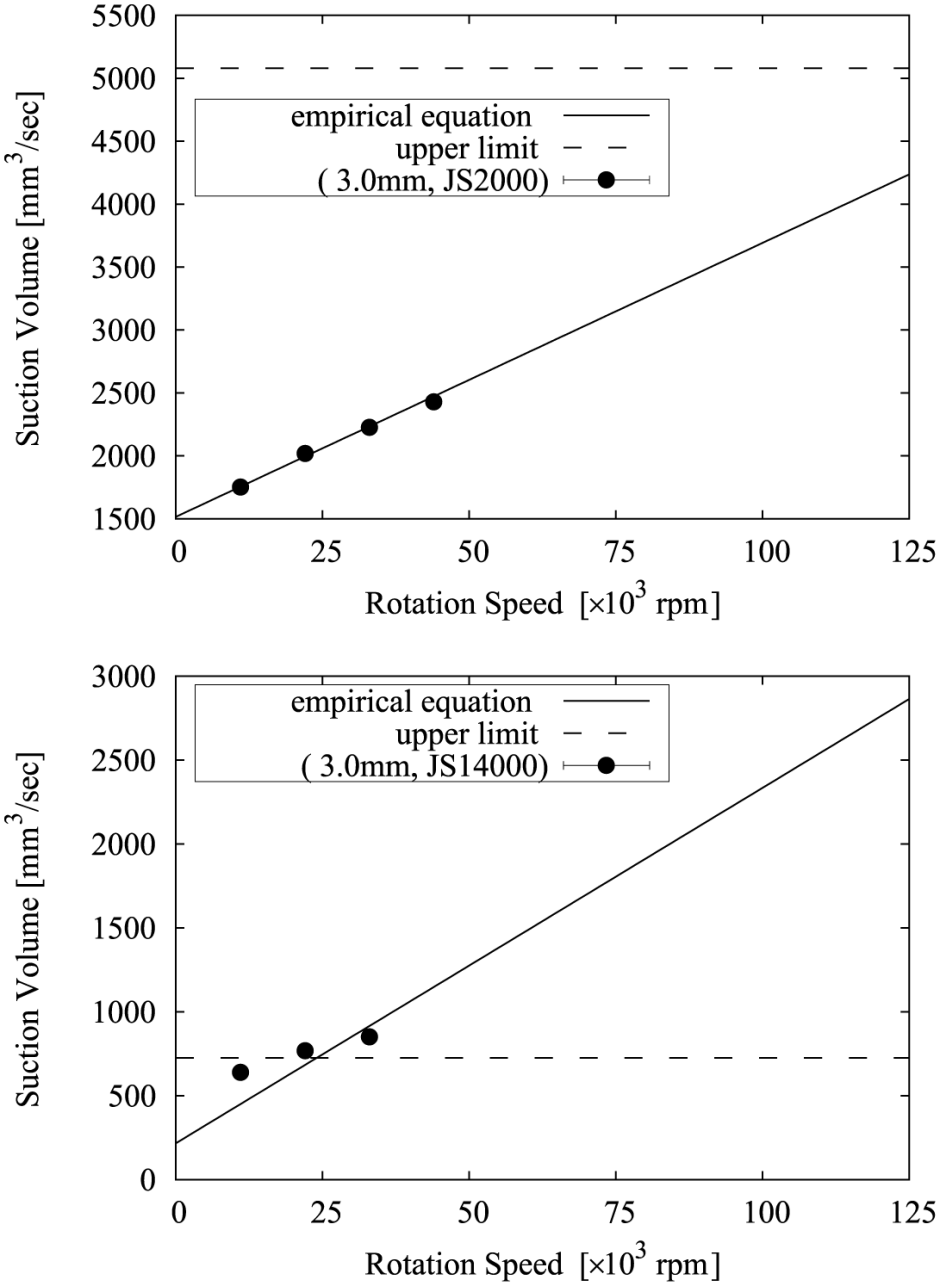
The relationship between Eqs 8 and 9 and the suction volume of the device with a 3.0 mm tip.

The relationship between Eqs 8 and 9 and the measurement results is shown in Figs 8, 9, 10, 11 and 12. Two approximate straight lines express the tendency of the suction volume *Q* versus the screw rotation speed *N* without contradiction. The suction volume is substantially proportional to rotation speed in the case in which the suction volume is sufficiently small versus *Q*_*upper*_ and is closer to *Q*_*upper*_ according to the increase of the rotation speed. However, the graph from the results with 0.8 mm and 1 mm tips for JS14000 is reduced after closing to *Q*_*upper*_. This is probably because the flow velocity around the axial center of the screw, with the force applied only in the rotating direction, and/or the flow velocity of the gap would become 0 or a minus value (reverse flow) because the volume conveyed by the screw can’t exceed to the input volume. Since, from Eq 5, the difference of force (velocity) between the inner and outer of the screw is dependent on the screw size, the suction volume of a 1 mm tip device tends to be decreased more rapidly than that of a 0.8 mm tip device under the same conditions. In addition, from the results of the device with a 0.8 mm tip for JS14000, JS52000 and JS160000, the difference between the measurement value and *Q*_*upper*_ became small according to the increase of the viscosity because the high viscosity reduces change in speed (acceleration/deceleration) of the flow and the leakage of the high viscosity liquid is small under the same condition.

Considering use of the proposed device for removal of diseased tissue and assuming it would be used to remove a target tissue which is about 50 cc (= 50×10^3^ mm^3^) in size at approximately 15 minutes, if the viscosity of the tissue corresponds to a fish sausage (= 3,000–30,000 mPa·sec), suction performance is required to be at least 50 mm^3^/sec and a device with a 2 or 3 mm tip is suitable. In addition, a maximum rotation speed of 30,000 rpm is satisfactory. If finer resection is necessary, a device with a 1 mm tip also can be a candidate. When the target is a very high viscosity tissues; i.e. the process is similar to mixing meat (> 200,000 mPa·sec), in order to suppress the flow volume through the device and reduce the resistance being applied to the screw, for example, the intermittent operation of the cutting and suction may be necessary.

In addition, from Eqs 8 and 9, the device shape, in particular the screw shape, can be optimized for the target, operative procedure, and controllability. For example, since the upper limit of the suction volume can be predicted, a suitable size of the device can be chosen in order to restrict the cutting and suction speed, or the adjustment range of the rotation speed can be expanded in order to control the cutting and suction amount.

Therefore, based on several conditions expected during use of the device, we are developing and optimizing a cutting edge shape of the screw to realize the cutting and suction of the target tissue.

## Conclusion

Our objective in this study was to develop a narrow-diameter, long-bore cutting and suction device for use in minimally invasive surgery. We developed a suction mechanism that uses a twist blade screw made by twisting a thin plate, and evaluated its performance.

Experiments on fluids of different viscosities showed that our proposed twist blade screw exhibited high levels of suction performance for highly viscous fluids even with a narrow-diameter, long-bore tube. Using the proposed device, efficient suction will be possible even if crushed tissue is expected to have a high viscosity.

From the results, the empirical equation as a design index of the device was obtained. We intend to apply this equation to optimize a device capable of simultaneous cutting and suction and we will evaluate its performance. We will then undertake a basic study using the device to cut body tissue and other similar soft solids and suction the fragments, with the aim of completing a device that is capable of efficiently cutting and suctioning simultaneously.

We are planning to conduct experiments in a simulated surgical environment to evaluate the effectiveness of our device from perspectives including its operability and invasiveness during surgery.

## Supporting Information

S1 Video The performance of the prototype device for cutting and suction of the fresh chicken meat as a target. (MP4)
